# Natural Pest Regulation and Its Compatibility with Other Crop Protection Practices in Smallholder Bean Farming Systems

**DOI:** 10.3390/biology10080805

**Published:** 2021-08-20

**Authors:** Baltazar J. Ndakidemi, Ernest R. Mbega, Patrick A. Ndakidemi, Philip C. Stevenson, Steven R. Belmain, Sarah E. J. Arnold, Victoria C. Woolley

**Affiliations:** 1 Department of Sustainable Agriculture, School of Life Sciences and Bioengineering, The Nelson Mandela African Institution of Science and Technology, Arusha P.O. Box 447, Tanzania; ernest.mbega@nm-aist.ac.tz (E.R.M.); patrick.ndakidemi@nm-aist.ac.tz (P.A.N.); s.e.j.arnold@greenwich.ac.uk (S.E.J.A.); 2 Natural Resources Institute, University of Greenwich, Chatham Maritime, Kent ME4 4TB, UK; P.C.Stevenson@greenwich.ac.uk (P.C.S.); S.R.Belmain@greenwich.ac.uk (S.R.B.); V.Woolley@greenwich.ac.uk (V.C.W.); 3 Royal Botanic Gardens, Kew, Richmond, Surrey TW9 3DS, UK

**Keywords:** biological control, chemical control, biopesticides, habitat manipulation, predators, parasitoids, *Aphis fabae*, *Maruca vitrata*

## Abstract

**Simple Summary:**

Bean production by smallholder farmers in sub-Saharan Africa is frequently constrained by insect pests, two of the most serious being *Maruca vitrata* and *Aphis fabae*. For many bean farmers, the options available to control these pests are limited. A few can access synthetic insecticides, but these have negative consequences for their health and the environment. Natural pest regulation (NPR) offers environmentally benign approaches for smallholders to manage bean pests. For example, here, we focus on biological control whereby beneficial organisms predate or parasitize the pests. Field studies show this is a feasible strategy for controlling *M. vitrata* and *A. fabae*. In particular, we highlight how compatible biological control is with other NPR options, such as the use of biopesticides (including plant extracts), resistant varieties, and cultural control. We recommend that smallholder farmers consider biological control alongside other NPR strategies for reducing the populations of *A*. *fabae* and *M. vitrata* in the common bean, increasing the yields and reducing the negative impacts of the synthetic pesticides.

**Abstract:**

Common bean (*Phaseolus vulgaris*) production and storage are limited by numerous constraints. Insect pests are often the most destructive. However, resource-constrained smallholders in sub-Saharan Africa (SSA) often do little to manage pests. Where farmers do use a control strategy, it typically relies on chemical pesticides, which have adverse effects on the wildlife, crop pollinators, natural enemies, mammals, and the development of resistance by pests. Nature-based solutions —in particular, using biological control agents with sustainable approaches that include biopesticides, resistant varieties, and cultural tools—are alternatives to chemical control. However, significant barriers to their adoption in SSA include a lack of field data and knowledge on the natural enemies of pests, safety, efficacy, the spectrum of activities, the availability and costs of biopesticides, the lack of sources of resistance for different cultivars, and spatial and temporal inconsistencies for cultural methods. Here, we critically review the control options for bean pests, particularly the black bean aphid (*Aphis fabae*) and pod borers (*Maruca vitrata*). We identified natural pest regulation as the option with the greatest potential for this farming system. We recommend that farmers adapt to using biological control due to its compatibility with other sustainable approaches, such as cultural tools, resistant varieties, and biopesticides for effective management, especially in SSA.

## 1. Introduction

The challenge for agriculture today and in the next thirty years is implementing sustainable and ideally carbon zero farming that is economically viable and resilient to future shocks, including the changing climate. This is particularly important in Africa, where 19.1% of the population was undernourished in 2019 [1], and farmers are increasingly negatively affected by climate change. Agriculture underpins most livelihoods in sub-Saharan Africa (SSA), because it provides food security, employment, and also contributes to an average of 15% of the total gross domestic product (GDP) [2,3]. Small-scale farming is common in SSA, but smallholder farms have been neglected by existing studies [4]. It is thought that it will be particularly challenging for smallholders to adopt sustainable intensification, as they are vulnerable to production risks such as climate change [5].

Legumes, including common beans (*Phaseolus vulgaris*), are frequently grown by smallholder farmers in SSA. Beans are a critically important component of healthy diets in SSA, because they are good sources of protein; vitamins; energy; and micronutrients, e.g., iron, zinc, thiamin, and folic acid [6,7], and have the added benefit of fixing nitrogen through rhizobial interactions [7]. Therefore, the common bean and other legumes are ideal crops for increasing food security, improving soil quality, and enhancing livelihoods through increased income in SSA [8]. However, the yield of these grain legumes is constrained by insect pests [9]. For example, the common bean has an average yield gap of 2.6 Mg ha^−1^ across Ethiopia, Kenya, and Tanzania [10]. Reducing this gap could significantly increase the food security in these areas. The insect legume pests that cause yield reductions in common beans across SSA include scarab beetles (*Schizonycha* spp.), foliage beetles (*Ootheca bennigseni* and *Ootheca mutabilis*), black bean aphids (*Aphis fabae* and *Aphis craccivora*), bean stem maggots (*Ophiomyia phaseoli* and *Ophiomyia spencerella*), bean pod borers (*Maruca vitrata* and *Helicoverpa armigera*), tobacco whiteflies (*Bemisia tabaci*) biotype B, the Southern green stink bug (*Nezara viridula*), and the storage pest bean bruchid (*Callosobruchus maculatus*) [11,12,13,14]. The use of pest control strategies for most farmers in SSA is limited by the factors related to monetary cost, a lack of knowledge, and limited research on different products [15]. Synthetic pesticides are expensive for smallholder farmers, but they also have negative impacts on human health, the environment [16], and nontarget organisms such as natural enemies and pollinators [17]. Thus, there is a need for effective alternatives to suppress crop pests [18]. Natural pest regulation (NPR) offers an alternative for pest management in SSA and is an essential component of an integrated pest management system. NPR is an approach to pest management that relies on beneficial insects and biological approaches. It includes practices that are sustainable and are best-suited to smallholder farmers such as habitat manipulation to enhance the contribution and use of biological control agents [19,20], biopesticides including botanical insecticides [21], breeding for host–plant resistances [22], pheromones for mating disruptions [23], and cultural control [24]. Although there has been much research on developing these sustainable pest management options, there are few field-ready options targeted at or developed for smallholder farmers, especially in SSA and particularly for *M. vitrata* and *A. fabae*, which cause major yield losses (Table 1). Here, we critically review the existing methods to control *A. fabae* and *M. vitrata* sustainably using biological control techniques and their compatibility with other sustainable control strategies. Recommendations regarding the future directions of research and effective management options for these pests are presented, and the current challenges in sustainable pest management faced by smallholder farmers, especially in SSA, are discussed in the context of available options.

## 2. Selected Common Bean Pests: Bean Pod Borer (*Maruca vitrata*) and Black Bean Aphid (*Aphis fabae*)

**Figure 1:**
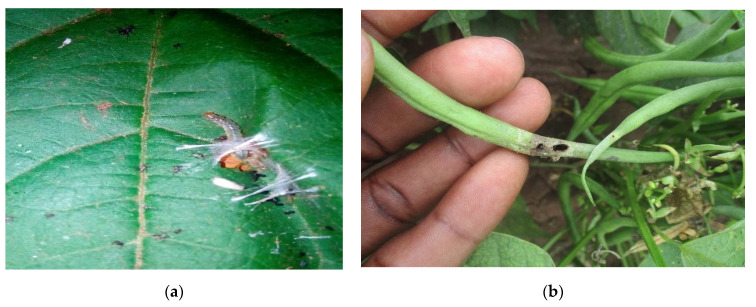
(**a**) *M. vitrata larva* and (**b**) pod damage by *M*. *vitrata* larva. *Maruca vitrata* infestation in beans (Photograph by Baltazar Ndakidemi, NM-AIST-Arusha, Tanzania).

**Figure 2:**
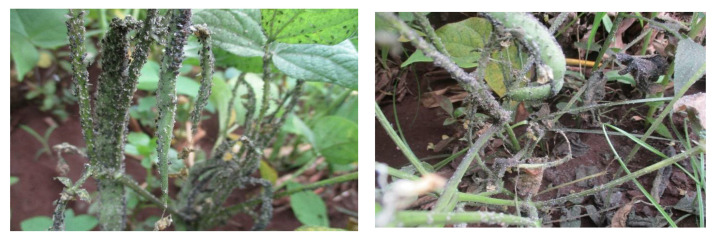
*Aphis fabae* infestation in beans (Photograph by Baltazar Ndakidemi, NM-AIST-Arusha, Tanzania).

*Maruca vitrata* and *A. fabae* (Figure 1 and Figure 2) are the pests of the greatest economic importance in beans, as they account for the major yield losses in SSA (Table 1). *Maruca vitrata* is a lepidopteran pest, the larvae of which reduce the yield by feeding on bean flowers, buds, and pods [31]. This pest is widely distributed in the tropics and subtropics and is highly destructive in many parts of Africa and Asia [32,33,34]. It has previously been recorded as causing between 15% and 53% yield losses in East African countries (Table 1). Another key pest of legumes in SSA is *A. fabae*, which causes damage by direct feeding; it is also responsible for the spread of several plant diseases, including cucumber mosaic virus (CMV), bean common mosaic necrosis virus (BCMNV) and bean common mosaic virus (BCMV) [35]. *Aphis fabae* can cause between 37% and 90% yield losses in East African countries (Table 1). Several host plants are associated with *A. fabae* and *M. vitrata* (see Table 2).

## 3. The Use of Biological Control as a Central Focus for *Aphis fabae* and *Maruca vitrata* Control

Due to the health and environmental hazards of synthetic pesticides, biological control has been proposed as an alternative in legumes [47]. Natural enemies can be an important components of integrated pest management (IPM) in agricultural fields and are environmentally benign compared with synthetic chemical pesticides and, in many cases, are economically viable [48]. IPM is a decision-based sustainable approach that utilizes all suitable pest management techniques (biological, cultural, physical/mechanical, and chemical methods) to reduce and/or manage pest populations, diseases, and weeds [49]. Thus, biological control is the process of NPR whereby natural enemies (predators and parasitoids) control populations of other plants and animals (e.g., insect pests) [50]. These beneficial organisms (also called biocontrol agents) control pests by different mechanisms, such as parasitism, predation, and competition [51]. There are three types of biological controls: conservation, classical, and augmentative [52]. Conservation biological control involves human interactions to enhance these natural enemies’ populations. For classical biological control, non-native natural enemies are released into areas where pests are invasive for permanent suppression, while augmentative biological control involves the mass rearing and release of native natural enemies for controlling pests [53,54,55]. A lack of knowledge about these control methods among farmers in SSA could be responsible for their lack of adoption [9].

Conservation biological control may be the most accessible form of biological control for smallholder farmers in SSA, because they can implement affordable field-scale interventions, such as increasing the local plant diversity and abundance, to enhance the natural enemy populations [56,57,58]. Predators/parasitoids can be attracted to crops through the provision of floral resources (nectar and pollen) in the vicinity and suitable habitats [59,60,61,62,63]. However, farmers’ knowledge of natural enemies and how to conserve them is a constraint to using this form of biological control [64]. More studies are required to identify the key roles played by natural enemies in managing important legume pests and their benefits over synthetics [9]. Natural enemies interact with field margins for resources and shelter, and this can enhance the biological control of pests in field crops [57,58]. This supports the importance of bean field margins in promoting natural enemies within bean crops. However, few field studies have investigated the impacts of different plant manipulations on the populations of natural enemies for suppressing pests such as *A. fabae* [65] and *M. vitrata* in legumes.

Several natural enemies have been identified for controlling *M. vitrata* at all developmental stages (Table 3). Both *M. vitrata* adult and larval predators have been investigated for their potential as effective biological control agents. The predators identified for *M. vitrata* include the Araneidae (*Nephila maculata*), Oxypidae (*Oxyopes javanus*), Anthocoridae (*Orius tantillus*), Forficulidae (*Diaperasticus erythrocephala*), and Formicidae (*Camponotus rufoglaucus*) [66]. Parasitoids of *M. vitrata* have been identified across a range of crops, including common beans and *Sesbania cannabina*. The hymenopteran larval parasitoids identified in SSA include *Braunsia kriegeri*, *Apanteles taragamae*, *Pristomerus* sp., *Bassus bruesi*, *Testudobracon* sp., *Cadurcia* sp., *Phanerotoma syleptae*, *Dolichogenidea* spp. and *Phanerotoma leucobasis* [67,68,69,70,71]. The egg parasitoids include *P. syleptae* and *Trichogramma* spp. [67,72,73,74]. Tachinid flies have also been identified as larval parasitoids [67,68].

The biological control of aphids is particularly difficult because of their high reproductive rates [76]. However, several aphid predators have been reported [77,78,79,80]. The predators of *A. fabae* (Table 4) include Coccinellidae; Cantharidae; Diptera (Dolichopodidae, Tachinidae, Syrphidae larvae, and Cecidomyiidae larvae); Staphylinidae; Hymenoptera (Vespidae and Polistinae); Hemiptera (Anthocoridae); and Neuroptera larva (Chrysopidae) [81,82,83,84,85]. Coccinellids are particularly effective predators of *A. fabae* [86,87]. *Hippodamia variegata* has been closely associated with *A. fabae* control in Kenya, while other predatory coccinellids associated with the control of *A. fabae* include *Cheilomenes* spp., *Henosepichna* spp. and *Exochomus* spp. [86]. Studies on *A. fabae* parasitoids have identified the braconid *Aphidius colemani* as the main primary parasitoid of *A. fabae* in Tanzania, although it is not yet known whether it is also present in other SSA countries [83,88].

When considering the use of natural enemies for pest control, it is crucial to understand how the biotic and abiotic factors influence them. For example, the rate of parasitism by *A. taragamae* decreases as the temperature increases, although this varies depending on the age of the larva. The first and second instars of *M. vitrata* larva are parasitized to a great extent, while the older larvae are not, which is likely due to the defensive behavior in older larvae [75]. Conversely, the parasitism by *A. colemani* decreases linearly with the temperature [90]. The competition for prey also can reduce parasitism [91]. These variables inform how best to deploy NPR in the field.

### Ecological Manipulations for Supporting Natural Enemies

Generally, conservation biological control utilizes plant and landscape biodiversity to promote beneficial insect populations by carefully modifying and managing the environment to increase non-prey resources [92,93,94,95,96]. Farmscaping is one term used to describe an ecological approach that enhances the biodiversity to augment the presence of beneficial organisms. Terms such as conservation biological control and ecological engineering are also used to describe similar interventions. Conservation biological control seeks to utilize the existing environmental components to support natural enemies [20]. Thus, ecological engineering and farmscaping are the strategies that are used in conservation biological control. Ecological engineering involves practices and interventions that aim to maximize the benefit of habitat management in suppressing agricultural pests [20]. Farmscaping provides suitable plants to support and attract populations of beneficial insects [97]. It forms the basis for ecological intensification or sustainable intensification, i.e., maximizing the ability of the system to produce food sustainably [98]. Farmscaping provides places for insects to overwinter, physical refugia, and forage; it may also act as a habitat for alternative prey and hosts [61,62,94,99]. Habitat management involving the manipulation of farmland vegetation can exert direct suppressive effects on pests and promote natural enemies [94]. Examples include the use of trap crops, hedgerows, field margins, and cover crops. Habitat disturbance, loss, and fragmentation in agroecosystems may lead to unsuitable environments for natural enemies [97]. Increasing landscape diversity through manipulating the plants that provide alternative resources to natural enemies such as pollen and nectar is therefore important for augmenting NPR and pollination [92,100]. A recent meta-analysis demonstrated a positive effect of flower strips on pest control compared to the fields without flower strips [101]. However, farmscaping might support pest populations, as some herbivores will depend on sugar and floral resources [102,103,104]. For instance, several studies reported that there was no effect of habitat manipulation on either pest or natural enemy abundances [105,106]. Therefore, it is vital to understand how natural enemies and pests interact with plants before assuming that increased diversity might improve the conservation biological control.

Several flowering plants found in SSA have been used in farmscaping. *Fagopyrum esculentum*, *Lobularia maritima*, *Mentha piperita*, *Tridax procumbens*, *Tagetes erecta*, *Tagetes minuta,* and *Sesamum indicum*, for example, have been found to increase the parasitism rates and longevity of lepidopteran and aphid’s natural enemies, such as *Apanteles ruficrus*, *Cotesia chilonis*, *Cotesia rubecula*, *Trichogramma chilonis*, *Microplitis mediator*, *Diadegma semiclausum,* and *A. colemani*; thus, they could have potential for *M. vitrata* and *A. fabae* management [106,107,108,109]. However, as some of these species are invasive to SSA, they should be used cautiously. Crops such as sorghum used as a border in pigeon pea fields increase the abundance of the predators *Coccinella septumpunctata*, *Cheilomenes sexmaculata,* and spiders, with a decrease in *M. vitrata* populations, which eventually leads to increased pigeon pea yields [110].

Farmscaping plants can also influence the behavior of phytophagous and predatory insects through the volatiles they produce [111,112]. Volatiles are involved in signaling and therefore play a part in the defense against various pathogens and herbivores and attract beneficial insects [113]. Intercropping some plants that release volatiles when damaged by herbivores will attract natural enemies of pests to an area. Some semiochemicals produced by plants when attacked by herbivores or synthetic equivalents will repel pests and attract natural enemies and are known as Herbivore-Induced Plant Volatiles (HIPVs) [114,115]. These can be used to improve the conservation biological control. For instance, volatiles released by damaged *M. vitrata* host plants (cowpea and pea bush) attracted the braconid *Therophilus javanus* and the parasitic fly *Nemorilla maculosa* [73,116]. This was also the case for volatile compounds produced by *Vicia faba* damaged by *A. fabae*, which attracted a parasitoid (*Lysiphlebus fabarum*) and a predator (*Orius albidipennis*) [117]. The use of synthetic HIPVs in attracting *M*. *vitrata* parasitoids was successful for *A. taragamae* and *P. syleptae* [118]. The mechanism of how HIPV functions could also be necessary for providing the control of *A. fabae,* and this requires further studies. Non-crop habitats such as field margins are essential in providing floral resources to natural enemies. Therefore, selecting suitable plants to attract/conserve natural enemies is essential in establishing the effective biological control of pests. Future studies should focus on different host plants and how they influence the abundance of insect pests and natural enemies and manipulate them for effective biological control.

## 4. Synthetic Chemicals and Their Impact on Natural Enemies of *Maruca vitrata* and *Aphis fabae*

Synthetic pesticides can provide the rapid knockdown of pests and reduce crop damage and yield losses when used correctly, but they also have negative impacts on human health and the environment, which can be a particular problem in SSA, where farmers often use older, more toxic, mislabeled pesticides that are more likely to be incorrectly handled and sprayed [119,120]. Issues such as mixing different types of chemicals and the increasing frequency and application rates further magnify the problems and create a need for environmentally friendly pest management [47]. In the context of biological pest control, pesticides can also kill nontarget organisms, such as the natural enemies of pests and pollinators, and so are detrimental to sustainable pest control [65].

The chemical control of *M. vitrata* is difficult, because their feeding sites in floral parts and pods protect the larvae from sprays [121,122], so NPR using biological pest control is more appropriate for this pest. Pesticide resistance to cypermethrin and dimethoate has also been reported in *M. vitrata*, making their control more challenging [123]. Farmers sometimes use doses of synthetic pesticides above the recommended rates to achieve control of *M. vitrata*, which increases nontarget impacts and risks and exacerbates the build-up of pesticide resistance [123]. Where farmers are provided knowledge and support, sustainable pest management strategies and a decreased reliance on synthetic pesticides are feasible [64].

Recent evidence has shown that a number of natural enemies of *A. fabae,* including Coccinellidae, Araneidae, Syrphidae, and Chrysopidae, were significantly lower in fields treated with the synthetic chemical pesticide Karate 5 EC (lambda-cyhalothrin) in common bean, cowpea, and pigeon pea field trials compared to those treated with botanicals [65,124,125]. Other studies have reported lethal and nonlethal effects of synthetic pesticides on arthropod natural enemies, such as feeding deterrents and mortality [126,127,128,129,130,131].

NPR that optimizes the services of natural enemies of *A. fabae* and *M. vitrata* is likely to be the basis of sustainable pest management. Since most synthetic pesticides are not compatible with beneficial insects, they will likely be used as the last resort in future sustainable farming systems. Thus, in searching for sustainable pest control, biological control stands as a cornerstone of other sustainable strategies and is favored for its feasibility [36], especially for smallholder farmers. However, smallholder farmers require knowledge support of biological control, as knowledge gaps hinder the adoption of conservation biological control [47,64]. Hence, it is important to make a proper advocacy of biological control for sustaining ecosystem services offered by the natural enemies of pests.

## 5. Other Sustainable Alternatives Compatible with Natural Enemies for Managing *Maruca vitrata* and *Aphis fabae*

### 5.1. Biopesticides

Biopesticides are natural products or microorganisms that act as alternatives to conventional pesticides and are nonpersistent in the environment, reducing the harmful effects of conventional pest control that rely on synthetic products [21,132,133,134,135]. Biopesticides are categorized into three groups: microbial pesticides, plant-incorporated protectants (PIPs), and biochemical pesticides or plant-based pesticides (PBPs) [136]. Some biopesticides can be used alone, while others can be coapplied with other control methods—in particular, the natural enemies of pests. Biopesticides can interact with other control options, especially natural enemies, and exhibit additive or antagonistic effects in their control of pests [137].

Microbial biopesticides consist of formulated microorganisms, including bacteria, fungi, protozoa, nematodes, and viruses, that are pathogenic to insects [132,136] and have been investigated for the management of *M. vitrata* and *A. fabae*. For example, *Bacillus thuringinesis* (Bt) and its toxins are one of the most widely used and successful microbial biopesticides [138] and have been successfully used to control *M. vitrata* based on the activity of Bt δ-endotoxins [139], while a commercial Bt product (Bactospeine) causes larval mortality [140]. The efficacy of Bt against *M. vitrata* has also been assessed in the field on yard-long beans, where its application in combination with PBPs such as neem is effective in reducing pod damage by *M. vitrata* [72]. However, the effects of these technologies on the natural enemies of pests are not well-studied and require more attention to determine the compatibility across sustainable approaches to pest management.

Viruses also have efficacy against *M. vitrata*, although most studies have been based in the laboratory [141]. The baculovirus, *M. vitrata* multi-nucleopolyhedrovirus (MaviMNPV), is a promising candidate as a biopesticide against *M. vitrata* and has been researched for use in SSA in particular. This virus causes significant mortality and reduces the egg viability of *M. vitrata* under laboratory conditions [142]. It has also been demonstrated to be effective against *M. vitrata* larva both in the field and in laboratory bioassays. Furthermore, its efficacy can be increased when applied with neem oil and neem, *Azadirachta indica,* and *Jatropha curcas* extracts [143,144,145], as evidence of its compatibility with other technologies. There have been attempts to develop techniques to mass produce MaviMNPV for smallholder farmers in SSA by the International Institute of Tropical Agriculture (IITA-Benin). Although this has not yet been implemented in smallholder farms on a large scale, it has the potential to control *M. vitrata* in a more cost-efficient and sustainable way [146]. Biopesticides for *A. fabae* and *M. vitrata* are shown in Table 5. The compatibility of microbial biopesticides with the natural enemies of pests has been reported. For instance, the baculovirus *M. vitrata* multi-nucleopolyhedrovirus (MaviMNPV) showed compatibility with the braconid parasitoid *A. taramagae*, and the parasitoid can transmit the virus between hosts [145].

Similarly, the entomopathogenic fungi (EPF) biopesticide *Beauveria bassiana* is compatible with the predatory coccinellids *C. septempunctata* and *H. variegata* [147,148], while *Lecanicillium muscarium*, in combination with the predatory coccinellid *Adalia bipunctata*, showed the possibility of reducing *A. fabae* infestations, although this was not in the field setting, and thus, further field trials are needed [149]. Likewise, a combination of the EPF *Metarhizium anisopliae* with the PBP pyrethrum does not affect the foraging behavior of *A. colemani*, a key parasitoid of *A. fabae*, implying a likely compatibility. However, when used alone, the EPF exhibited a deterrent effect in *A*. *colemani*, so more research is needed [150].

Although biopesticides, including EPFs, *B. thuringiensis*, entomopathogenic nematodes, and baculovirus-based products that target pests other than *M. vitrata* and *A. fabae*, are available commercially in SSA countries such as Kenya [146] and Tanzania [135], the knowledge of their production, the high cost of buying them, and their low speed of killing pests have made farmers reluctant to adopt these control options [135]. The efficacy of biopesticides may be altered by factors such as the humidity, rainfall, temperature, ultraviolet light, leaf surface chemistry, formulation, application method, substrate, and fungal isolates, meaning that their inappropriate use can give poor results [157,158]. Therefore, all these factors are to be considered in the formulation for increasing the efficacy of biopesticides for the sustainable control of pests.

Many EPFs affect specific orders of arthropods differently and may therefore pose a lower risk to natural enemies than target pests and, so, can be used effectively with natural enemies [159,160]. Conversely, some biopesticides, such as the EPF *B. bassiana*, infect and kill parasitoids such as *A. colemani* [161], and therefore, toxicity assays are critical in understanding the wider consequences of applying biopesticides for managing pests.

PBPs exploit naturally occurring entomotoxins of plant origin. They contain metabolites that can inhibit and kill directly, affect reproduction, and alter other metabolic processes in pests [136,162]. PBPs can be highly toxic but have typically had lower environmental impacts than synthetic pesticides, primarily because they do not persist in the environment and are broken down through the actions of sunlight and microorganisms [65,124]. Thus, the longer-term impacts on natural enemies and biological control are reduced. The potential of PBPs has been explored in some African countries. However, few of these pesticides have been exploited commercially or in smallholder farms, although some, such as pyrethrum and neem, have been developed into highly successful commercial products [163]. PBPs are compatible with natural enemies and can potentially be used in SSA [124,125]. Many PBPs have significantly reduced the negative impacts on beneficial invertebrates compared to synthetic chemical pesticides [124,125]. For example, extracts of *Bidens pilosa*, *Lippia javanica*, *Tephrosia vogelii*, *Lantana camara*, *Vernonia amygdalina,* and *Tithonia diversifolia* were highly effective at controlling insect pests on legume crops, but their impact on lady beetles, lacewings, spiders, and syrphid flies was reportedly significantly lower than those resulting from exposure to synthetic pesticides [21,65,124,125]. However, several studies outside SSA have also shown that some PBPs exhibit a toxicity to the natural enemies of pests. For instance, pyrethrum, neem, Chilean plant products, and rotenone caused the mortality of the natural enemies *Adonia variegata*, *Venturia canescens*, *Orius laevigatus,* and *Encarsia formosa* [164,165,166,167].

PBPs, including commercially available products such as Neem Baan (Azadirachtin), have been found to cause *M. vitrata* larval mortality, although their efficacy is reduced when applied to the later larval stages [140]. Due to the cost of commercial PBPs, it may be more viable for smallholder farmers in SSA to use extracts (Table 6) that are prepared by themselves and that are readily available and accessible [134,168]. For instance, multiple pesticidal plant extracts from common margin plants and weeds have shown efficacy against *A*. *fabae*, including *A. indica*, *Allium sativum*, *Eucalyptus* sp., *Swietenia* sp., *Tephrosia vogelii* [169,170], *Matricaria chamomilla* on the broad bean, and tobacco water (*Nicotiana* sp.) on yard-long beans [171]. Additionally, *Annona muricata* and *Piper guineense* extracts significantly reduced *M. vitrata* larval infestations and, thus, increased cowpea yields compared with the synthetic pesticide lambda-cyhalothrin in cowpeas [172].

Many more plant extracts and their compatibility with natural enemies have not yet been investigated. Additionally, the use of plant products to control *M. vitrata* is practically limited, because little research has been conducted on PBPs targeting *M. vitrata*, despite using some of these plants in managing *A. fabae* [65,124,125]. As the *M*. *vitrata* larva feeds inside the pods, flowers, and flower bud and also webs the leaves and flowers, it is protected from spraying with PBPs as much as with synthetics, making foliar sprays difficult to apply effectively [31]. The lack of field trials and field data, particularly for legume crops in Africa, and the toxicity of some PBPs are the drawbacks of effective pest management. Thus, further studies should address these aspects for the effective control of bean pests. 

Plant-incorporated protectants (PIPs) are biopesticides such as the gene encoding the Bt toxin that can be introduced into the plant genome that allows the plant to produce this toxin and increases the resistance of the plant to some pests [178]. For instance, the Cry proteins expressed in Bt cowpeas have shown a less negative effect on nontarget organisms [179]. Generally, the PIPs available globally for the specific management of *M. vitrata* and *A. fabae* are limited, and equally, their impacts on their natural enemies have not been studied; thus, more work has to be done to address this gap.

There is considerable effort for biopesticides to be used more widely in SSA for pest control. However, studies have shown that there is generally a lack of awareness of biopesticide products among smallholder farmers. Across Uganda and Kenya, fewer than 20% of the farmers surveyed had heard of biopesticides [180], and in Kenya, only 10% of the farmers surveyed had used biopesticides on their crops [181]. The lack of information could be one of the key factors preventing the broader use of biopesticides across SSA. Other potential barriers to the uptake of biopesticides include the production, cost of buying them, concern over their speed of killing pests, and short persistence. Expertise on product development, packaging, and composition; the knowledge gap among smallholder farmers; and research on the importance and benefits of biopesticides such as ecological safety and the possession of nutrient supplements that are advantageous to crops should be addressed [134].

### 5.2. Use of Resistant Varieties with Natural Enemies

The use of resistant varieties is an important component of IPM in controlling legume pests [182]. The use of resistant varieties has shown some level of effectiveness in legumes such as cowpeas [183]. Some *M*. *vitrata*-resistant varieties have been identified [184,185,186]. Fewer studies have been conducted on the use of resistant varieties to reduce *M. vitrata* infestations in the common bean, and therefore, this requires more work, especially considering the impacts on nontarget species that might be exposed to these toxins through parasitizing or by predating the pests. For *A. fabae* management, effective, resistant varieties of *P. vulgaris* have been identified both inside [187] and outside [188] SSA. Cultivars of *V. faba* and *Beta vulgaris* resistant to *A. fabae* have similarly been identified [189,190]. Resistant cultivars have been used in combination with the natural enemies of bean pests for better pest control. For example, the combined effect of using partially resistant cultivars of *V. faba* with the coccinellid predator *C. septempunctata* was effective in the control of *A. fabae* [189]. However, studies to test the different resistant bean varieties and their compatibility with the natural enemies of bean pests for maximizing the biological control services in bean fields are needed.

### 5.3. Cultural Control

Control practices such as mixed cropping systems, variations in planting dates, plant density, and spacing can be effective against *A. fabae* and *M. vitrata*; furthermore, they are beneficial for natural enemies [24,131,191,192]. The low incidence of *M*. *vitrata* larva and flower pod damage was observed in intercropping systems compared to monocropping systems in some studies [193,194]. However, several other studies showed no evidence of the use of intercropping systems in reducing the *M. vitrata* effects [195,196]. Thus, it is important to generate information on the limitations and opportunities of employing intercropping approaches in combating *M. vitrata,* especially in common beans, because currently, there are limited studies on this, especially where they enhance the natural enemy benefits.

Cultural practices may not always benefit pest control. For example, manipulations and variations in planting dates, weeding, and spacing did not help in reducing *M. vitrata* populations but, rather, increased the pest populations [191,197]. Contrarily, a previous study showed that early cowpea planting reduces the rates of infestations by *M. vitrata* [198]. For *A. fabae*, it was observed that intercropping *P. vulgaris* with *Zea mays* was successful in reducing aphid populations in *P. vulgaris* [199]. Currently, limited studies have been conducted to examine the applicability of different cultural practices for *A. fabae* management in *P. vulgaris*. Therefore, it is of importance to assess the potential of different cultural practices in combination with natural enemies and the biological control of bean pests to reduce *A. fabae* infestations in common bean fields.

The compatibility of cultural practices with natural enemies of *M. vitrata* and *A. fabae* has been reported. The assessment of the intercropping of cowpeas with either green gram or sorghum revealed a high abundance of predators (coccinellids, hoverflies, rove beetles, spiders, praying mantis, ground beetles, predatory mites, big-eyed bugs, dragonflies, damsel bugs, minute pirate bugs, and earwigs) [131]. Conversely, intercropping sorghum with a cowpea crop did not produce significant differences in *M. vitrata* populations, although the parasitism rate by *P. leucobasis* was higher in the intercropping plots [185]. The coccinellid populations (*H*. *variegata* and *C*. *septempunctata*), important natural enemies of *A*. *fabae*, were enhanced by intercropping faba beans with the aromatic flowering plant dragonhead (*Dracocephalum* spp.) [200]. However, limited studies have been conducted to evaluate the compatibility of other cultural control practices with the natural enemies of *A. fabae* and *M. vitrata,* particularly in beans.

## 6. Conclusions and Recommendations

Synthetic pesticides are still the most frequently used management strategy to control *M. vitrata* and *A. fabae* by smallholder farmers in bean farming systems, yet are largely incompatible with a biological control that is dependent on beneficial insects. Here, we reviewed more sustainable approaches to the pest management of these pests in beans and compared their compatibility with other natural pest-regulating options. Ultimately, there is inadequate evidence to indicate how suitable different approaches might be from the existing literature, and this needs to be addressed. However, where there is evidence, this strongly supports the notion that more sustainable pest management interventions are more compatible with biological control than synthetic pesticides (Table 7). The deployment of sustainable approaches for *M*. *vitrata* and *A*. *fabae* management for biopesticides faces challenges. For example, the use of biopesticides is limited by the lack of field trials, and thus, farmers have a poor perception of their effectiveness, high cost of production, challenges facing their registration, and hence, few biopesticides are available commercially. More field trials, increasing awareness, and the registration of new products at affordable prices are strategies that would enable farmers in SSA to use more biopesticides.

Additionally, the incorporation of biological control into management strategies for smallholders is exacerbated by low levels of experience, expertise, and broader knowledge about the natural enemies among smallholder farmers and their requirements, especially how landscapes can influence the abundance and diversity of biological control species. In some cases, the species that support beneficial insects may also provide botanical pesticides, such as *Tephrosia vogelii*, *Annona muricata,* and *Piper guineense*, and a combination of control options with biological control have shown potential in decreasing *M. vitrata* and *A. fabae* infestations while achieving high yields compared with synthetic pesticides [170,172,191]. However, the yield data for different management options are also lacking, and therefore, it is of importance to address this gap. The synergistic effects of *M. vitrata* and *A. fabae* control are exhibited when some biopesticides are used with natural enemies. Therefore, more studies should focus on different biopesticides and their compatibility with natural enemies for increasing the efficacy in the management of *A. fabae* and *M. vitrata*. The use of IPM has been advocated in the literature as a solution to sustainably managing pests for years [201,202], but the implementation still requires work and, in particular, more compatibility studies.

A combination of strategies such as the cultural control, application of biopesticides, and using resistant varieties with the efficient monitoring of pests could work together with natural enemies to bring about the effective management of *M. vitrata*. Some works have reported an increasing control efficiency when a combination of strategies such as resistant cowpea varieties; cultural methods (manipulating of the planting date, adjusting planting spacing, and using mixed cropping); and the reduced application of synthetic pesticides with natural enemies was used in IPM [36,137,203,204]. One challenge that limits the efficacy of using a cultural control is its dependence on seasons and locations. Additionally, the lack of sources of resistance traits has limited the development of some resistant varieties for *M. vitrata* and *A. fabae*. The use of resistant varieties and cultural control could help in enhancing the efficacy of *M. vitrata* and *A. fabae* controls, and thus, more studies should explore the control of these pests by using biological controls with resistant varieties and cultural control efficacy to minimize the losses by *M. vitrata* and *A. fabae*.

This literature review discussed biological control as a feasible IPM strategy for smallholder farmers of SSA. The evidence indicates a considerable potential in employing available plant diversities from farmers’ localities to manage pests while enhancing the populations of natural enemies for *M. vitrata* and *A. fabae* management. This form of NPR is also compatible with other control strategies, such as biopesticides, cultural control, and the use of resistant crop varieties. However, the use of biopesticides requires more investment in their development and efficacy in controlling *M. vitrata* and *A. fabae*. Equally, more work is needed in developing resistant varieties for *M. vitrata* and *A. fabae*; some of the identified varieties for controlling *M. vitrata* and *A. fabae* in the surveyed literature have been found to exhibit a moderate resistance to *M. vitrata* and *A. fabae*. The use of cultural control for *M. vitrata* has shown inconsistent results, and thus, there is a need to generate more knowledge on the limitations and potential of this approach as part of an integrated system.

A community-based approach to implementing different management alternatives, such as the growth/preparation of PBPs, the production of EPF/viruses at a community level, or the knowledge exchange among smallholder farmers about natural enemies, would help to overcome some of these issues [15,64,205]. The training and capacity building of farmers could also facilitate a better understanding of biological control and its compatibility with other control strategies for enhancing the ecosystem services provided by natural enemies. The information reviewed here informs how biological control is a key component in the sustainable management of *A. fabae* and *M. vitrata* and how it interacts with other sustainable approaches to the management network of key bean pests. More knowledge should be generated on the importance of a system-based ecological approach to increase the understanding of the management options.

## Figures and Tables

**Table 1:** The empirically assessed bean yield losses attributed to black bean aphid (*Aphis fabae*) and bean pod borer (*Maruca vitrata*) in East African countries per year.

Bean Pest	Country	Yield Loss%	Reference
*A. fabae*	Burundi	50	[25]
*A. fabae*	Kenya	37–90	[26]
*A. fabae*	Tanzania	37	[27]
*A. fabae*	Uganda	90	[28]
*M. vitrata*	Tanzania	33–53	[29]
*M. vitrata*	Kenya	15–25	[30]

**Table 2:** Host plant species for *Maruca vitrata* and *Aphis fabae*.

Bean Pest	Plant Species	Family	References
*M. vitrata*	*Vigna unguiculata*	Fabaceae	[36,37]
*M. vitrata, A fabae*	*Phaseolus vulgaris*	Fabaceae	[36]
*M. vitrata*	*Cajanus cajan*	Fabaceae	[31]
*M. vitrata, A. fabae*	*Phaseolus lunatus*	Fabaceae	[31,38,39]
*M. vitrata*	*Sesbania* sp.	Fabaceae	[40]
*M. vitrata*	*Crotalaria* sp.	Fabaceae	[41]
*M. vitrata*	*Sesbania pachycarpa*	Fabaceae	[42]
*M. vitrata, A. fabae*	*Vicia faba*	Fabaceae	[31,43]
*A. fabae*	*Beta vulgaris*	Amaranthaceae	[44,45]
*A. fabae*	*Solanum tuberosum*	Solanaceae	[46]
*A. fabae*	*Allium cepa*	Amaryllidaceae	[39]
*A. fabae*	*Lycopersicon esculentum*	Solanaceae	[46]
*A. fabae*	*Dahlia pinnata, Lactuca sativa*	Asteraceae	[39,46]

**Table 3:** Parasitoids of *Maruca vitrata* that have been reported in SSA.

Parasitoid Species	Family	Host Stage Parasitized	References
*Apanteles taragamae*	Braconidae	Larva	[75]
*Bassus bruesi, Bracon* sp.	Braconidae	Larva	[68]
*Braunsia kriegeri*	Braconidae	Larva	[67,68]
*Cadurcia* sp.	Tachinidae	Larva	[68]
*Dolichogenidea* sp.	Braconidae	Larva	[68,70]
*Phanerotoma leucobasis*	Braconidae	Larva	[58]

**Table 4:** Natural enemies for black bean aphids (*Aphis fabae*), also recorded in SSA.

Natural Enemy	Family	References
*Aphidius colemani*	Aphididae	[83,88]
*Cheilomenes* sp.	Coccinellidae	[86]
*Exochomus* spp.	Coccinellidae	[86]
*Henosepichna* spp.	Coccinellidae	[86]
*Hippodamia variegata*	Coccinellidae	[86,89]

**Table 5:** Microbial biopesticides used in the control of *Aphis fabae* and *Maruca vitrata*.

Bean Pest	Biopesticide Used	References
*M. vitrata*	*Bacillus thuringiensis*	[140]
*M. vitrata*	*Beauveria bassiana*	[141,145,151]
*M. vitrata*	Mavi multi-nucleopolyhedrovirus	[141,142]
*M. vitrata*	*Metarhizium anisopliae*	[151,152]
*A. fabae*	*Lecanicillium muscarium*	[153,154]
*A. fabae*	*Simplicillium lamellicola*	[153,154]
*A. fabae*	*Aspergillus flavus*	[155]
*M. vitrata*	*Heterorhabditis* sp., *Oscheius* sp.	[156]

**Table 6:** Pesticidal plants common to SSA used for different pest controls.

Pesticidal Plant	Family	Family	References
*Ageratum conyzoides*	Asteraceae	*Tribolium castaneum*	[173]
*Allium sativum*	Amaryllidaceae	*Aphis fabae*	[169]
*Annona muricata*	Annonaceae	*Maruca vitrata*	[172]
*Azadirachta indica*	Meliaceae	*Aphis fabae*	[169]
*Eucalyptus* sp.	Myrtaceae	*Aphis fabae*	[169]
*Euphorbia heterophylla*	Euphorbiaceae	*Sitophilus zeamais*	[174]
*Matricaria chamomilla*	Asteraceae	*Aphis fabae*	[172]
*Ocimum* sp.	Lamiaceae	*Didyctium* sp.	[175]
*Parthenium hysterophorus*	Asteraceae	*Aphis craccivora*	[176]
*Piper guineense*	Piperaceae	*Maruca vitrata*	[172]
*Swietenia* sp.	Meliaceae	*Aphis fabae*	[169]
*Tephrosia purpurea*	Fabaceae	*Odoiporus longicollis*	[177]
*Tephrosia vogelii*	Fabaceae	*Aphis fabae*	[124,170]

**Table 7:** The compatibility of the *Maruca vitrata* (MV) and *Aphis fabae* (AF) management options with their natural enemies.

Biological Control Agent	Control Method													
	Biopesticides								Resistant Varieties		Cultural Control		Synthetic Pesticides	
													(−)	
	EPF		Bacteria		Viruses		Botanical							
							Pesticides							
	AF	MV	AF	MV	AF	MV	AF	MV	AF	MV	AF	MV	AF	MV
Predators	(+)	(*)	(*)	(*)	(*)	(*)	(+)	(*)	(+)	(*)	(+)	(+)	(−)	(*)
	[145,148,149]						[21,65,124,125]		[189]		[131,200]	[131]	[65,124,125]	
	(+)	(*)	(*)	(*)	(*)	(+)	(+)	(*)	(*)	(*)	(*)	(+)	(*)	(*)
Parasitoids	[150]					[145]	[150]					[185]		

Note: (+) shows compatibility; (−) shows non-compatibility; (*) implies the compatibility is less-studied.

## Data Availability

Not applicable.
